# Identification of a Heritable Polymorphism in Bovine *PRNP* Associated with Genetic Transmissible Spongiform Encephalopathy: Evidence of Heritable BSE

**DOI:** 10.1371/journal.pone.0002912

**Published:** 2008-08-13

**Authors:** Eric M. Nicholson, Brian W. Brunelle, Juergen A. Richt, Marcus E. Kehrli, Justin J. Greenlee

**Affiliations:** 1 Virus and Prion Diseases of Livestock Research Unit, National Animal Disease Center, USDA, Agricultural Research Service, Ames, Iowa, United States of America; 2 Pre-Harvest Food Safety and Enteric Diseases Research Unit, National Animal Disease Center, USDA, Agricultural Research Service, Ames, Iowa, United States of America; Leiden University Medical Center, Netherlands

## Abstract

**Background:**

Bovine spongiform encephalopathy (BSE) is a transmissible spongiform encephalopathy (TSE) of cattle. Classical BSE is associated with ingestion of BSE-contaminated feedstuffs. H- and L-type BSE, collectively known as atypical BSE, differ from classical BSE by displaying a different disease phenotype and they have not been linked to the consumption of contaminated feed. Interestingly, the 2006 US H-type atypical BSE animal had a polymorphism at codon 211 of the bovine prion gene resulting in a glutamic acid to lysine substitution (E211K). This substitution is analogous a human polymorphism associated with the most prevalent form of heritable TSE in humans, and it is considered to have caused BSE in the 2006 US atypical BSE animal. In order to determine if this amino acid change is a heritable trait in cattle, we sequenced the prion alleles of the only known offspring of this animal, a 2-year-old heifer.

**Principal Findings:**

Sequence analysis revealed that both the 2006 US atypical BSE animal and its 2-year-old heifer were heterozygous at bovine prion gene nucleotides 631 through 633 for GAA (glutamic acid) and AAA (lysine). Both animals carry the E211K polymorphism, indicating that the allele is heritable and may persist within the cattle population.

**Conclusions:**

This is the first evidence that the E211K polymorphism is a germline polymorphism, not a somatic mutation, suggesting BSE may be transmitted genetically in cattle. In the event that E211K proves to result in a genetic form of BSE, this would be the first indication that all 3 etiologic forms of TSEs (spontaneous, hereditary, and infectious) are present in a non-human species. Atypical BSE arising as both genetic and spontaneous disease, in the context of reports that at least some forms of atypical BSE can convert to classical BSE in mice, suggests a cattle origin for classical BSE.

## Introduction

Transmissible spongiform encephalopathies (TSEs) are a class of neurodegenerative diseases that affect various mammals. They are caused by abnormally folded prion proteins that induce the conversion of the non-infectious, cellular form of the host prion protein (PrP^C^) into the abnormal, infectious form (PrP^Sc^) [1]. In humans, TSEs can be acquired through exposure to infectious material, inherited as germline polymorphisms in the prion gene (*PRNP*), or occur spontaneously. In other mammals, exposure to infectious material is the only confirmed natural route.

Bovine spongiform encephalopathy (BSE), a TSE of cattle, can be subdivided into at least three groups: classical, H-type, and L-type, with the latter 2 collectively referred to as atypical BSE. Susceptibility or resistance to a TSE disease can be influenced by at least 3 factors related to the host prion protein: protein expression levels, the number of octapeptide repeats, and specific amino acid differences. These 3 factors are all relevant to prion biology in cattle. Non-coding region polymorphisms in cattle have been identified that modulate expression level [2], [3] and influence susceptibility to classical bovine spongiform encephalopathy (BSE) [4], [5] but not atypical BSE [6]. The presence of additional octapeptide repeats in transgenic mice [7], [8] and Brown Swiss cattle [9], [10] have been reported to result in increased susceptibility to classical BSE. Amino acid differences are a major component in susceptibility and resistance to acquired TSE disease in sheep [11] and are the basis for genetic TSEs in humans [12]. The most common amino acid substitution in humans associated with inherited Creutzfeldt-Jakob Disease (CJD) is at codon 200 in which a glutamic acid is replaced with a lysine (E200K). The presence of the lysine at this codon results in complete penetrance of CJD [13].

It was determined that *PRNP* in the 2006 US H-type BSE case had an encoded amino acid change at bovine codon 211 (E211K) in one allele, a change that is analogous to the human E200K substitution [14]. To date, no other coding region polymorphism analogous to a polymorphism associated with a genetic TSE in humans has been identified in cattle [12], [15], nor has this polymorphism been previously reported in any other cattle despite the sequencing efforts of multiple studies involving several thousand cattle. No parental information for the 2006 US atypical BSE case is available, so it is unknown if the E211K polymorphism was inherited or caused by a spontaneous mutation. As part of an epidemiologic investigation targeted toward identification of animals of the birth cohort of the 2006 US H-type BSE case 2 farms were identified as having contained the animal and 35 other farms identified as having potentially supplied the animal to those farms. Despite this investigation, the source herd was not identified providing no means to ascertain heritability by analysis of siblings of this animal [16]. To address if the E211K polymorphism is a heritable trait, we tested the only known living offspring of the 2006 US atypical BSE positive animal, a 2-year-old heifer [16]. If the offspring encodes the same E211K polymorphism, then the allele is heritable and is possibly circulating in the bovine population.

## Materials and Methods

Extraction, amplification and sequencing of DNA were carried out as previously described [6]. The 2-year old heifer was housed under an approved National Veterinary Services Laboratory animal care and use committee protocol. DNA from this animal was extracted from both blood and buccal swab tissue using the High Pure PCR Template Preparation Kit (Roche Applied Science, Indianapolis, IN). For the 2006 atypical BSE animal, brain tissue was collected in the field following euthanasia by a veterinarian attending to the downer animal. DNA was extracted from the brain tissue as previously described [17]. Polymerase chain reaction primer pairs were used to amplify a 986-bp region encompassing the PRNP coding region in cattle, and internal sequencing primers provided four-fold coverage of the gene. To ensure accuracy of the results, each DNA sample was subsequently re-amplified and sequenced. Sequences for the 2006 atypical BSE animal and the 2-year-old heifer were submitted to GenBank (accession numbers EU557971 and EU557972 respectively).

## Results

**Figure 1:**
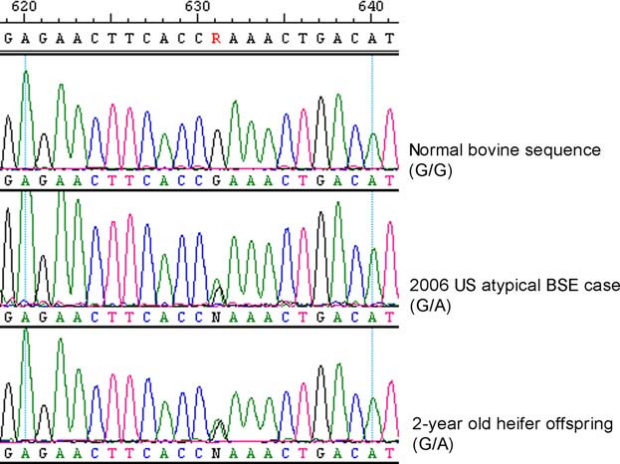
Sequencing output for bovine reference sequence, 2006 US atypical BSE case, and 2-year-old heifer offspring. The bovine reference sequence is homozygous for G at nucleotide 631. The 2006 US atypical BSE case and the 2-year-old heifer offspring are heterozygous G/A at nucleotide 631.

In the bovine prion gene, codon 211 corresponds to nucleotides 631 through 633, which are typically homozygous GAA/GAA in cattle and encodes glutamic acid in both alleles. Our sequence analysis confirmed the 2006 atypical BSE animal was heterozygous GAA/AAA for these nucleotides where the AAA codon encodes a lysine. We also established that the 2-year-old heifer is likewise heterozygous GAA/AAA for nucleotides 631 through 633. Output from DNA sequencing depicting the presence of the heterozygous codon for these two animals, as well as a normal bovine reference sample, are shown in Figure 1.

## Discussion

Few genetic polymorphisms within the bovine *PRNP* coding region result in an amino acid change [15], and only these two related animals encode for an amino acid substitution analogous to any polymorphism associated with an inherited TSE. While rare, the fact that the substitution is heritable clearly indicates that this allele may be present in the cattle population at some, albeit low, frequency.

Etiology of TSEs is complex with human TSEs known to occur as infectious, spontaneous, and genetic diseases. While transmission through peripheral exposure to infectious material is clearly involved in the feedborne, classical BSE epizootic, the origin of BSE is unresolved. Although sheep scrapie has been suggested as a potential source of BSE [18], scrapie has yet to be transmitted to cattle orally [19]. This discrepancy necessitates a reevaluation of the source of the BSE epizootic. Single amino acid substitutions made within mouse PrP analogous to those associated with human genetic TSEs were found to cause comparable disease in transgenic mice [20]–[22]. Thus, the identification of the E211K polymorphism in cattle has important implications. Here we report a germline polymorphism present in cattle analogous to a polymorphism in humans that results in a genetic TSE with complete penetrance.

The fact that the 2-year-old heifer has not developed clinical signs associated with BSE is consistent with the late onset of hereditary human TSEs [23] and atypical BSE [24]. Consistent with these observations, the 2006 US atypical BSE animal that contained the E211K variant was estimated to be 10 years of age at onset of clinical signs [16]. The heifer will be used to generate experimental animals containing the E211K polymorphism for studies that will establish if the polymorphism influences TSE susceptibility in cattle and if the E211K polymorphism results in genetic BSE. Recently, it has been suggested that the atypical BSE cases that lack amino acid changes in the prion protein are likely spontaneous TSEs of cattle akin to spontaneous TSEs in humans [6], [24]–[26]. The presence of the E211K polymorphism in an atypical BSE positive animal, however, indicates a potential genetic etiology of cattle TSEs much like inherited TSEs in humans. This preliminary evidence of a genetically based cattle TSE suggests for the first time that all 3 etiologic forms of TSEs (spontaneous, hereditary, and infectious) may be present in a non-human species. Atypical BSE arising as both genetic and spontaneous disease, in the context of data indicating L-type BSE can convert to classical BSE in mice [27], suggests a cattle origin for classical BSE. This could have occurred either spontaneously or as a result of a polymorphism, such as E211K, that was then amplified by the practice of feeding these affected bovine tissues to cattle. This feeding practice has since been banned in the United States and other nations. Because of the spontaneous and inherited etiology of BSE, eradication is not an attainable goal and a low incidence of BSE can be expected to persist. Compliance with the existing ruminant to ruminant feed ban should, however, prevent another BSE epizootic comparable to that which occurred in the U.K.

## References

[1] Prusiner SB (1998). Prions.. Proc Natl Acad Sci USA.

[2] Kashkevich K, Humeny A, Ziegler U, Groschup MH, Nicken P (2007). Functional relevance of DNA polymorphisms within the promoter region of the prion protein gene and their association to BSE infection.. Faseb J.

[3] Sander P, Hamann H, Drogemuller C, Kashkevich K, Schiebel K (2005). Bovine prion protein gene (*PRNP*) promoter polymorphisms modulate *PRNP* expression and may be responsible for differences in bovine spongiform encephalopathy susceptibility.. J Biol Chem.

[4] Juling K, Schwarzenbacher H, Williams JL, Fries R (2006). A major genetic component of BSE susceptibility.. BMC Biol.

[5] Sander P, Hamann H, Pfeiffer I, Wemheuer W, Brenig B (2004). Analysis of sequence variability of the bovine prion protein gene (*PRNP*) in German cattle breeds.. Neurogenetics.

[6] Brunelle BW, Hamir AN, Baron T, Biacabe AG, Richt JA (2007). Polymorphisms of the prion gene promoter region that influence classical bovine spongiform encephalopathy susceptibility are not applicable to other transmissible spongiform encephalopathies in cattle.. J Anim Sci.

[7] Castilla J, Gutierrez-Adan A, Brun A, Pintado B, Parra B (2004). Different behavior toward bovine spongiform encephalopathy infection of bovine prion protein transgenic mice with one extra repeat octapeptide insert mutation.. J Neurosci.

[8] Castilla J, Gutierrez-Adan A, Brun A, Pintado B, Salguero FJ (2005). Transgenic mice expressing bovine PrP with a four extra repeat octapeptide insert mutation show a spontaneous, non-transmissible, neurodegenerative disease and an expedited course of BSE infection.. FEBS Lett.

[9] Geldermann H, He H, Bobal P, Bartenschlager H, Preuss S (2006). Comparison of DNA variants in the PRNP and NF1 regions between bovine spongiform encephalopathy and control cattle.. Anim Genet.

[10] Sauter-Louis C, Clauss M, Chaher E, Klee W, Wichmann HE (2006). Breed predisposition for BSE: epidemiological evidence in Bavarian cattle.. Schweiz Arch Tierheilkd.

[11] Baylis M, Goldmann W (2004). The genetics of scrapie in sheep and goats.. Curr Mol Med.

[12] Mead S (2006). Prion disease genetics.. Eur J Hum Genet.

[13] Spudich S, Mastrianni JA, Wrensch M, Gabizon R, Meiner Z (1995). Complete penetrance of Creutzfeldt-Jakob disease in Libyan Jews carrying the E200K mutation in the prion protein gene.. Mol Med.

[14] Clawson ML, Richt JA, Baron T, Biacabe AG, Czub S (2008). Association of a bovine prion gene haplotype with atypical BSE.. PLoS ONE.

[15] Seabury CM, Honeycutt RL, Rooney AP, Halbert ND, Derr JN (2004). Prion protein gene (*PRNP*) variants and evidence for strong purifying selection in functionally important regions of bovine exon 3.. Proc Natl Acad Sci USA.

[16] USDA-APHIS (2006). Alabama BSE Investigaiton Final Epidemiology Report May 2, 2006.. http://www.aphis.usda.gov/newsroom/hot_issues/bse/downloads/EPI_Final5-2-06.pdf.

[17] Richt JA, Kunkle RA, Alt D, Nicholson EM, Hamir AN (2007). Identification and characterization of two bovine spongiform encephalopathy cases diagnosed in the United States.. J Vet Diagn Invest.

[18] Arnold ME, Wilesmith JW (2004). Estimation of the age-dependent risk of infection to BSE of dairy cattle in Great Britain.. Prev Vet Med.

[19] Cutlip RC, Miller JM, Hamir AN, Peters J, Robinson MM (2001). Resistance of cattle to scrapie by the oral route.. Can J Vet Res.

[20] Hsiao KK, Groth D, Scott M, Yang SL, Serban H (1994). Serial transmission in rodents of neurodegeneration from transgenic mice expressing mutant prion protein.. Proc Natl Acad Sci U S A.

[21] Hsiao KK, Scott M, Foster D, Groth DF, DeArmond SJ (1990). Spontaneous neurodegeneration in transgenic mice with mutant prion protein.. Science.

[22] Telling GC, Haga T, Torchia M, Tremblay P, DeArmond SJ (1996). Interactions between wild-type and mutant prion proteins modulate neurodegeneration in transgenic mice.. Genes Dev.

[23] Horwich AL, Weissman JS (1997). Deadly conformations–protein misfolding in prion disease.. Cell.

[24] Baron T, Biacabe AG (2006). Origin of bovine spongiform encephalopathy.. Lancet.

[25] Biacabe AG, Laplanche JL, Ryder S, Baron T (2004). Distinct molecular phenotypes in bovine prion diseases.. EMBO Rep.

[26] Casalone C, Zanusso G, Acutis P, Ferrari S, Capucci L (2004). Identification of a second bovine amyloidotic spongiform encephalopathy: molecular similarities with sporadic Creutzfeldt-Jakob disease.. Proc Natl Acad Sci USA.

[27] Capobianco R, Casalone C, Suardi S, Mangieri M, Miccolo C (2007). Conversion of the BASE Prion Strain into the BSE Strain: The Origin of BSE?. PLoS Pathog.

